# High hepatitis C virus infection among female sex workers in Viet Nam: strong correlation with HIV and injection drug use

**DOI:** 10.5365/wpsar.2019.10.1.002

**Published:** 2019-07-25

**Authors:** Linh-Vi N Le, Siobhan O’Connor, Tram Hong Tran, Lisa Maher, John Kaldor, Keith Sabin, Hoang Vu Tran, Quang Dai Tran, Van Anh Thi Ho, Tuan Anh Nguyen

**Affiliations:** a Kirby Institute for Infection and Immunity, UNSW Sydney, Australia.; b United States Centers for Disease Control and Prevention, Atlanta, GA, United States of America.; c National Institute of Hygiene and Epidemiology, Hanoi, Viet Nam.; d UNAIDS, Geneva, Switzerland.; e Partners in Health Research, Hanoi, Viet Nam.; f General Department of Preventive Medicine, Ministry of Health, Hanoi, Viet Nam.; g United States Centers for Disease Control and Prevention, Hanoi, Viet Nam.

## Abstract

**Objective:**

The World Health Organization’s guidelines on viral hepatitis testing and treatment recommend prioritizing high prevalence groups. Hepatitis C virus (HCV) infection disproportionately affects people who inject drugs and men who have sex with men, but data on female sex workers (FSW) are limited. The study aimed to determine active HCV infection and risk factors associated with HCV exposure among Vietnamese FSW.

**Methods:**

We surveyed 1886 women aged ≥ 18 years from Haiphong, Hanoi and Ho Chi Minh City who had sold sex in the last month. We tested for HCV antibody and HCV core antigen as markers for exposure to HCV and active infection, respectively.

**Results:**

Across these provinces, high prevalence of HCV exposure (8.8–30.4%) and active infection (3.6–22.1%) were observed. Significant associations with HCV exposure were HIV infection (aOR = 23.7; 95% CI: 14.8–37.9), injection drug use (aOR = 23.3; 95% CI: 13.1–41.4), history of compulsory detention (aOR = 2.5; 95% CI: 1.4–4.2) and having more than 10 sex clients in the last month (aOR = 1.9; 95% CI: 1.2–3.2). Among FSW who reported never injecting drugs, HIV infection (aOR = 24.2; 95% CI: 14.8–39.4), a history of non-injection drug use (aOR = 3.3, CI: 1.8–5.7), compulsory detention (aOR = 2.2; 95% CI: 1.2–4.0) and having over 10 sex clients in the last month (aOR = 2.2, 95% CI: 1.3–3.7) were independently associated with HCV exposure.

**Discussion:**

FSW have elevated HCV risks through sex- and drug-related pathways. These findings highlight the need to offer FSW-targeted HCV interventions and ensure their access to HIV prevention and treatment.

Globally, an estimated 71 million people were living with chronic hepatitis C virus (HCV) infection, and 1.75 million were newly infected with HCV in 2015. (*1*) Approximately 75% of acute HCV infections result in chronic infections. In the absence of treatment, 15–30% of people develop cirrhosis within 20 years with subsequent increased risk of hepatocellular carcinoma and death. (*2*) The estimated number of new HCV infections exceeds the estimated number of deaths and cures together, (*1*) warranting rapid scale-up of both preventive and therapeutic interventions for viral hepatitis.

The primary mode of HCV transmission is blood contact, frequently by reuse of injecting equipment or other skin-piercing devices. (*3*) Accordingly, 23% of new HCV infections occur in people who inject drugs (PWID), and 31% of deaths from chronic HCV infections are attributable to a history of injection drug use. (*4*) Interventions for prevention have focused on use of sterile equipment for skin-piercing procedures, including making clean equipment available to PWID. Direct-acting antiviral (DAA) medications providing effective cures for HCV infection are yet to become widely available in many low- and middle-income countries. (*1*) Until there is universal access to DAAs, national viral hepatitis responses may have to prioritize certain populations for testing and treatment; World Health Organization (WHO) guidelines recommend targeting populations with evidence of higher chronic HCV prevalence. (*5*)

Greater understanding of the viral hepatitis epidemic is needed as global and national responses set strategic directions and priorities. Countries of Asia account for one third of global chronic HCV infections. (*1*) In Viet Nam, mathematic modelling estimated 1 million people to be living with chronic HCV and 24 300 new annual HCV infections, 11% of which are attributed to injection drug use and 60% to blood transfusions or medical services. (*6*) Recent studies in Viet Nam documented high HCV infection prevalence among PWID, with up to 80% of PWID exposed to the virus. (*7*, *8*) More recently, injection drug use also correlated with HCV infection among Vietnamese men who have sex with men (MSM). (*9*) There is an assumption that female sex workers (FSW) have an elevated risk for blood-borne and sexually transmitted infections, potentially through dual sexual and drug use transmission pathways, but there is limited information on HCV in this population globally, including in Viet Nam. The current study aimed to address the global data gap for knowledge about HCV infection among FSW to inform the viral hepatitis response.

## Methods

### Data collection

Viral hepatitis investigation was added to the 2013 round of the routine Integrated Biological and Behavioural Surveillance (IBBS) conducted by the Viet Nam National Institute of Hygiene and Epidemiology (NIHE) and the United States Centers for Disease Control and Prevention (US CDC). We conducted separate cross-sectional surveys among street-based sex workers (SSW) and venue-based sex workers (VSW) from Hanoi, Haiphong and Ho Chi Minh City (HCMC) using time-location sampling from June to October 2013. The survey consisted of interviews and HCV serological testing. Women aged ≥ 18 years who sold sex in the last month (*n* = 1886) were eligible to be recruited. The sample sizes were calculated to detect 20% anti-HCV prevalence and expected prevalence based on the 2009 IBBS surveys (*10*) with 95% confidence level, 2–5% targeted confidence interval width, and adjustment for design effect. For each province and population, we generated a sampling frame of locations where FSW were known to be present. Locations for the SSW sampling frame were streets, parks and other openly public spaces; venues for the VSW sample were entertainment or service establishments.

The number of sex workers present at each location was enumerated through field visits. Locations were randomly selected with probability proportional to size. At each selected cluster, invitational coupons were provided to potential participants to enroll in the study conducted at health clinics. Sample sizes were reached for all populations.

Women who fulfilled the eligibility criteria and provided written voluntary informed consent underwent individual face-to-face interviews conducted by trained interviewers. A structured questionnaire used in previous IBBS rounds was used to collect information on sociodemographic characteristics, sex work history, condom use behaviours, alcohol and drug use risks, sex partner drug use risks, incarceration history and access to health services. A series of questions were asked about different types of illicit drugs, including lifetime and recent drug use, injection drug use and sharing of needles and syringes. Certified laboratory technicians collected venous blood by venipuncture. No personal identifying information was collected. The study protocol and data collection instruments were reviewed and approved by the Ethics Review Board of NIHE and the Internal Review Board of the US CDC.

### Laboratory tests

The participants’ serum was tested using the Abbott ARCHITECT® automated immunologic assay platform and commercially available ARCHITECT assay kits for HCV antibody (anti-HCV) and HCV core antigen (HCVcoreAg), hepatitis B surface antigen (HBsAg), hepatitis B e antigen (HBeAg), and hepatitis B core antibodies (anti-HBc) (Abbott Laboratories, USA). Specimens were initially screened for anti-HCV and HBsAg and with subsequent testing of reactive specimens for HCVcoreAg and HBeAg, respectively. Each run included standardized ARCHITECT® controls.

Blood specimens were tested for HIV per national guidelines: screening for HIV antibody using Genscreen Ultra HIV Ag/Ab (Bio-Rad, USA) with confirmation of positive tests by Determine HIV-1/2 (Alere, Japan) and Murex HIV Ag/Ab Combination (DiaSorin, United Kingdom of Great Britain and Northern Ireland) testing. The National Reference Laboratory at NIHE conducted external quality assurance on a randomly selected 10% of HIV-negative and 5% of HIV-positive screening samples.

### Statistical analyses

Summary statistics were calculated by province and subpopulations. Sampling weights were applied to adjust for sampling probabilities in prevalence estimation. All statistical analyses were performed using STATA version 14, (*11*) with adjustments for correlation between and within clusters.

HCV infection status was classified as HCV exposure (anti-HCV-positive), active HCV infection (anti-HCV-positive and HCVcoreAg-positive) or no evidence of HCV exposure (anti-HCV-negative). For hepatitis B virus (HBV), classifications were past or active HBV infection (HBsAg-positive or anti-HBc-positive), active HBV infection (HBsAg-positive) and HBeAg-active HBV infection (HBsAg-positive and HBeAg-positive).

Univariable and multivariable analyses were conducted to determine factors associated with HCV exposure. We used Pearson’s χ2 statistic to test the association between selected demographics and risk characteristics and HCV exposure. Random effects logistic regression were used to account for intracluster correlation. Independent variables associated with HCV infection at *P* < 0.20 were entered into the multivariable logistic regression model and retained in the reduced model if association was *P* < 0.05 for the Wald statistic. Age and marital status were retained in the reduced model as a priori confounders. The variable “type of sex work” was defined based on participants’ responses to where they mainly negotiated sex rather than using recruitment venues. We calculated the population attributable risk (PAR) to show potential impact of injection drug use on infection prevalence. Records that were missing outcome variables were excluded.

## Results

### Sample characteristics and risk behaviours

**Table 1** presents sociodemographic and behavioural indicators by province and subpopulation of FSW. Across the subpopulations and provinces, the mean ages ranged from 28.9 to 35.4 years, and durations of sex work ranged from 4.9 to 7.4 years with SSW being older and working in the sex industry for a longer period of time than VSW. Formal education beyond the sixth grade was attained by a large majority (> 85%) of FSW in Hanoi and Haiphong and by lower proportions in HCMC. The median total monthly income was higher for VSW (US$ 430–480) than SSW (US$ 290–380). A history of drug use was high across all subpopulations, ranging from 8.3% to 31.8%. A history of injection drug use reached 24.2% among Haiphong SSW and ranged from 3.0% to 8.2% among other subpopulations.

**Table 1:** Demographic characteristics and HIV, HBV and HCV prevalence among female sex workers in three urban provinces of Viet Nam, 2013

Indicators	Street-based sex workers (*n* = 918)			Venue-based sex workers (*n* = 968)		
-	Hanoi	Haiphong	Ho Chi Minh City	Hanoi	Haiphong	Ho Chi Minh City
-	***n* = 299**	***n* = 204**	***n* = 415**	***n* = 300**	***n* = 304**	***n* = 364**
**Age (mean years) (s.d.)**	**32.7 (6.9)** *n* **= 299**	**34.6 (5.5)** *n* **= 204**	**35.4 (10.2)** *n* **= 413**	**29.0 (5.9)** *n* **= 300**	**28.9 (5.9)** *n* **= 304**	**29.0 (7.0)** *n* **= 363**
**Education (%)**	*n* **= 299**	*n* **= 204**	*n* **= 415**	*n* **= 300**	*n* **= 304**	*n* **= 363**
**Primary or no formal schooling (** ≤ **5)**	**14.4**	**14.2**	**50.2**	**8.0**	**4.6**	**29.2**
**Secondary school (6–9)**	**50.2**	**56.9**	**38.8**	**45.3**	**59.2**	**49.6**
**High school (10–12)**	**34.4**	**25.5**	**10.8**	**41.3**	**33.6**	**19.3**
**College/University (13+)**	**1.0**	**3.4**	**0.2**	**5.3**	**2.6**	**1.9**
**Years in sex work (mean) (s.d.)**	**7.3 (4.4)** *n* **= 293**	**7.0 (4.9)** *n* **= 169**	**7.4 (6.7)** *n* **= 402**	**6.0 (4.4)** *n* **= 290**	**4.9 (4.2)** *n* **= 260**	**5.1 (4.7)** *n* **= 357**
Median total monthly income (million VND*) (IQR)	**8.0** **(6.0–10.0)** *n* **= 299**	**6.0** **(5.0–7.2)** *n* **= 203**	**6.0** **(4.0–9.0)** *n* **= 386**	**10.0** **(8.0–12.0)** *n* **= 300**	**10.0** **(8.0–15.0)** *n* **= 303**	**9.0** **(6.0–12.0)** *n* **= 345**
**Consistent condom use with clients in past month (%) (95% CI)**	**82.3** **(76.2, 87.1)** *n* **= 299**	**76.5** **(65.6, 84.7)** *n* **= 204**	**60.2** **(53.4, 66.7)** *n* **= 415**	**75.7** **(68.4, 81.7)** *n* **= 300**	**87.2** **(81.6, 91.2)** *n* **= 304**	**57.1** **(51.0, 63.1)** *n* **= 364**
**Consumed at least one alcoholic drink daily in past month (%) (95% CI)**	**4.0** **(1.9, 8.2)** *n* **= 299**	**1.5** **(0.5, 4.0)** *n* **= 204**	**3.9** **(2.5, 5.9)** *n* **= 415**	**13.3** **(9.1, 19.2)** *n* **= 300**	**1.0** **(0.3, 3.0)** *n* **= 304**	**41.5** **(33.3, 50.1)** *n* **= 364**
**Ever used illicit drugs (%) (95% CI)**	**13.8** **(9.8, 17.7)** *n* **= 298**	**31.8** **(25.3, 38.4)** *n* **= 198**	**18.2** **(13.6, 22.8)** *n* **= 413**	**8.3** **(5.0, 13.7)** *n* **= 300**	**20.8** **(15.8, 26.8)** *n* **= 289**	**9.6** **(6.5, 14.0)** *n* **= 363**
**Ever injected any illicit drug (%) (95% CI)**	**6.4** **(3.8, 10.6)** *n* **= 298**	**24.2** **(16.1, 34.9)** *n* **= 198**	**8.2** **(5.7, 11.7)** *n* **= 415**	**5.3** **(2.9, 9.5)** *n* **= 300**	**7.6** **(4.1, 13.8)** *n* **= 289**	**3.0** **(1.5, 6.1)** *n* **= 363**
**Had a sex partner that injected drugs in past month (%) (95% CI)**	**17.7** **(14.1, 22.1)** *n* **= 299**	**20.6** **(14.0, 29.2)** *n* **= 204**	**15.7** **(12.2, 19.9)** *n* **= 415**	**12.0** **(8.3, 17.0)** *n* **= 300**	**11.5** **(7.7, 16.9)** *n* **= 304**	**8.3** **(5.5, 12.2)** *n* **= 363**
**Past and current HCV infection (%) (95% CI)**	**14.8** **(8.9, 23.4)** *n* **= 298**	**30.4** **(23.0, 38.9)** *n* **= 204**	**15.6** **(11.0, 21.7)** *n* **= 392**	**13.5** **(8.2, 21.2)** *n* **= 299**	**12.4** **(8.5, 17.7)** *n* **= 304**	**8.8** **(5.6, 13.6)** *n* **= 364**
**Active HCV infection (%) (95% CI)**	**8.4** **(4.6, 14.9)** *n* **= 298**	**22.1** **(15.8, 30.0)** *n* **= 204**	**9.4** **(6.7, 13.1)** *n* **= 392**	**5.6** **(3.4, 9.2)** *n* **= 299**	**8.2** **(5.4, 12.4)** *n* **= 304**	**3.6** **(2.0, 6.2)** *n* **= 364**
**Past and current HBV infection (%) (95% CI)**	**57.4** **(51.4, 63.2)** *n* **= 298**	**55.9** **(48.5, 63.0)** *n* **= 204**	**59.2** **(53.9, 64.4)** *n* **= 392**	**75.0** **(69.4, 79.8)** *n* **= 299**	**84.1** **(78.8, 88.2)** *n* **= 304**	**55.8** **(49.2, 62.3)** *n* **= 364**
**Active HBV infection (%) (95% CI)**	**8.4** **(5.7, 12.1)** *n* **= 298**	**7.4** **(4.5, 11.9)** *n* **= 204**	**8.8** **(6.1, 12.5)** *n* **= 392**	**8.9** **(6.2, 12.6)** *n* **= 299**	**11.1** **(7.5, 16.0)** *n* **= 304**	**5.6** **(3.1, 9.8)** *n* **= 364**
**HBeAg+ active HBV infection (%) (95% CI)**	**4.0** **(2.3, 7.0)** *n* **= 298**	**3.4** **(1.6, 7.1)** *n* **= 204**	**3.1** **(1.6, 5.8)** *n* **= 392**	**2.5** **(1.3, 4.9)** *n* **= 299**	**1.4** **(0.6, 3.4)** *n* **= 304**	**3.0** **(1.5, 5.8)** *n* **= 364**
**HIV infection (%) (95% CI)**	**10.4** **(6.2, 16.9)** *n* **= 298**	**31.9** **(22.5, 42.9)** *n* **= 204**	**13.5** **(10.1, 17.9)** *n* **= 415**	**16.0** **(11.1, 22.5)** *n* **= 300**	**10.5** **(6.6, 16.4)** *n* **= 304**	**8.2** **(5.3, 12.6)** *n* **= 364**
**Active HCV and HIV infections (%) (95% CI)**	**5.4** **(2.7, 10.5)** *n* **= 298**	**13.7** **(7.9, 22.9)** *n* **= 204**	**6.4** **(4.2, 9.6** *n* **= 392**	**5.4** **(3.2, 8.9)** *n* **= 299**	**5.3** **(3.1, 8.8)** *n* **= 304**	**1.6** **(0.6, 4.2)** *n* **= 364**
**Past and current HCV infection among FSW who were HIV-negative and reported never having injected drugs (%) (95% CI)**	**6.0** **(3.2, 11.0)** *n* **= 252**	**14.2** **(8.8, 22.0)** *n* **= 113**	**4.0** **(2.4, 6.7)** *n* **= 323**	**6.2** **(3.0, 12.4)** *n* **= 242**	**4.9** **(2.9, 8.1)** *n* **= 113**	**1.2** **(0.5, 3.2)** *n* **= 327**

CI = confidence interval, HBV = hepatitis B virus, HCV = hepatitis C virus, FSW = female sex workers, IQR = interquartile range, SD = standard deviation, VND = Vietnamese dong.

* Exchange rate at time of study = 21 0000 Vietnamese dong: US$ 1.

In the multivariable analysis (**Table 2**), over two fifths reported negotiating sex mainly on the street or in other public areas, and three quarters had sold sex to over 10 clients in the past month. Consistent condom use, defined as condom use for every vaginal or anal sex act with clients in the past month, was reported by 71.7%. Eight per cent of FSW had ever injected drugs, and 10.2% had been involuntarily detained in a rehabilitation centre.

**Table 2:** Association between HCV exposure and selected characteristics of female sex workers in three urban provinces of Viet Nam, 2013

Variable	%	Crude association		Adjusted association*	
		OR	95% CI	aOR	95% CI
*Sociodemographic*					
**Age (years)**	**(*n* = 1 858)**	**-**	**-**	**-**	**-**
**18–24**	**20.7**	**1.00**	**-**	**1.00**	**-**
**25–29**	**22.3**	**1.75**	**1.08, 2.81**	**1.13**	**0.59, 2.14**
**30–34**	**25.9**	**2.94**	**1.80, 4.81**	**1.28**	**0.67, 2.45**
**35+**	**31.1**	**2.12**	**1.29, 3.46**	**0.98**	**0.50, 1.94**
**Obtained grade 6 or higher education (%)**	**77.8** **(***n* **= 1 860)**	**1.14**	**0.82, 1.60**	**-**	**-**
**Marital status**	**(***n* **= 1 804)**	**-**	**-**	**-**	**-**
**Never been married**	**33.7**	**1.00**	**-**	**1.00**	**-**
**Married**	**12.6**	**1.02**	**0.63, 1.64**	**0.59**	**0.29, 1.19**
**Separated/divorced**	**46.6**	**1.44**	**1.03, 2.02**	**1.34**	**0.81, 2.22**
**Widowed**	**7.1**	**2.32**	**1.33, 4.04**	**1.59**	**0.74, 3.39**
***Occupational characteristics and sexual behaviours***					
**Age at first sex (per year increase)**	**(***n* **= 1 769)**	**0.94**	**0.89, 0.99**	**-**	**-**
**Years in sex work (per year increase)**	**(***n* **= 1 749)**	**1.03**	**1.02, 1.06**	**-**	**-**
**Total monthly income (VND, using IQR categories)**	**(***n* **= 1 811)**	**-**	**-**	**-**	**-**
** < 5 million**	**-**	**1.00**	**-**	**-**	**-**
**5 to < 7 million**	**-**	**0.75**	**0.50, 1.12**	**-**	**-**
**7 to < 8.5 million**	**-**	**0.71**	**0.45, 1.12**	**-**	**-**
≥ **8.5 million**	**-**	**0.75**	**0.51, 1.10**	**-**	**-**
**Negotiate sex mainly on street or in other public areas**	**43.1** **(***n* **= 1 860)**	**1.90**	**1.33, 2.73**	**-**	**-**
**Ever sold sex in other provinces**	**7.3** **(***n* **= 1 860)**	**1.66**	**1.06, 2.59**	**-**	**-**
**Had over 10 sex clients in past month**	**74.7** **(***n* **= 1 861)**	**2.33**	**1.55, 3.49**	**1.94**	**1.17, 3.21**
**Consistent condom use with clients in past month**	**71.7** **(***n* **= 1 861)**	**1.01**	**0.74, 1.36**	**-**	**-**
***Alcohol and drug use behaviours***					
**Daily alcohol consumption in past month**	**12.0** **(***n* **= 1 861)**	**0.68**	**0.41, 1.13**	**-**	**-**
**Injection drug use (lifetime)**	**8.1** **(***n* **= 1 836)**	**30.73**	**18.94, 49.87**	**23.32**	**13.13, 41.44**
***Sex partners’ drug use behaviour***					
**Sex partners injected drugs in past month**	**(***n* **= 1 860)**	**-**	**-**	**-**	**-**
**No**	**61.2**	**1.00**	**-**	**-**	**-**
**Yes**	**13.9**	**2.75**	**1.90, 3.97**	**-**	**-**
**Unknown**	**24.9**	**2.14**	**1.53, 3.01**	**-**	**-**
***Incarceration history***					
**Ever detained in rehabilitation centre for sex workers**	**10.2** **(***n* **= 1 853)**	**3.80**	**2.64, 5.49**	**2.46**	**1.44, 4.21**
***STI***					
**Experienced genital pain or ulcers in past 12 months**	**54.5** **(***n* **= 1 861)**	**1.29**	**0.97, 1.71**	**-**	**-**
***Blood-borne infections***					
**HBV**	**65.1** **(***n* **= 1 861)**	**0.82**	**0.63, 1.07**	**-**	**-**
**HIV**	**13.6** **(***n* **= 1 861)**	**18.47**	**13.27, 25.71**	**23.67**	**14.79, 37.88**
***Province***	**(***n* **= 1 861)**	**-**	**-**	**-**	**-**
**Hanoi**	**-**	**1.32**	**0.84, 2.12**	**-**	**-**
**Hai Phong**	**-**	**1.95**	**1.34, 2.86**	**-**	**-**
**Ho Chi Minh City**	**-**	**1.00**	**-**	**-**	**-**

* *n*  **= 1828. Variables in multivariate model are adjusted for age and marital status.**

### Prevalence of infection

HCV, HBV and HIV prevalence are presented in **Table 1**. Twenty-four specimens for the HCMC SSW population and one specimen for the Hanoi VSW were missing HBV and HCV results. Exposure to HCV infection was highest among Haiphong SSW (30.4%) and lowest among HCMC VSW (8.8%). In the remaining provinces and subpopulations, HCV exposure ranged from 12.4% to 15.6%. The prevalence of active HCV infection was highest among Haiphong SSW (22.1%) and lowest among HCMC VSW (3.6%) and ranged from 5.6% to 9.4% in the remaining provinces and subpopulations.

Across the provinces and subpopulations, HBV exposure ranged from 55.9% to 84.1%, whereas active HBV infections ranged from 5.6% to 11.1%. HBeAg-positive HBV infection ranged from 1.4% to 4.0%. There were no active HBV and HCV coinfections.

HIV prevalence patterns were similar to HCV, with the highest observed among Haiphong SSW (31.9%) and lowest among HCMC VSW (8.2%). Coinfection with HCV and HIV ranged from 1.6% among HCMC SSW to 13.7% among Haiphong SSW.

### Predictors of HCV exposure

In univariable analyses (**Table 2**), sociodemographic factors associated with anti-HCV prevalence included older age and being separated, divorced or widowed. Occupational characteristics showing significant crude associations were longer duration of sex work, negotiating sex mainly on the streets, selling sex in more than one province, having over 10 clients in the last month and ever having been confined in compulsory detention centres for sex workers. The sexual and drug use risk behaviours crudely associated with HCV exposure were ever using injection drugs and having a sex partner who had injected drugs in the last month or whose drug use status was unknown to the FSW. HIV infection was a strong predictor of HCV infection exposure.

When adjusted for age and marital status in multivariable analysis, HIV seropositivity and injection drug use were the strongest predictors of HCV exposure (**Table 2**). FSW who tested HIV-positive had 23.7 (95% CI: 14.8–37.9) times greater odds of having been exposed to HCV than those who tested HIV-negative. Compared to FSW who had never injected drugs, those who had ever injected drugs had 23.3 (95% CI: 13.1–41.4) times the odds of HCV infection exposure. The PAR for ever having injected drugs was 64.3% (95% CI: 49.4–76.5). Other independent predictors for HCV exposure were having over 10 sex clients in the last month and a history of compulsory detention.

HIV seropositivity is also the strongest predictor of HCV exposure in the subpopulation of FSW who self-reported never having injected drugs (**Table 3**). Other factors significantly associated with HCV were lifetime non-injection drug use, compulsory detention and having over 10 sex clients in the last month.

**Table 3:** Association between HCV exposure and selected characteristics of female sex workers who reported never having injected drugs in three urban cities of Viet Nam, 2013

Variable	%	Crude association		Adjusted association*	
		OR	95% CI	aOR	95% CI
***Sociodemographic***					
**Age (years)**	**(***n* **= 1 684)**	**-**	**-**	**-**	**-**
**18–24**	**21.4**	**1.00**	**-**	**1.00**	**-**
**25–29**	**22.2**	**1.98**	**1.09, 3.58**	**1.53**	**0.74, 3.16**
**30–34**	**25.0**	**3.24**	**1.73, 6.07**	**1.61**	**0.76, 3.41**
**35+**	**31.3**	**2.72**	**1.50, 4.94**	**1.63**	**0.76, 3.52**
**Obtained grade 6 or higher education (%)**	**(***n* **= 1 686)** **77.2**	**1.17**	**0.76, 1.82**	**-**	**-**
**Marital status**	**(***n* **= 1 686)**	**-**	**-**	**-**	**-**
**Never been married**	**34.0**	**1.00**	**-**	**1.00**	**-**
**Married**	**12.3**	**0.94**	**0.52, 1.68**	**0.53**	**0.24, 1.18**
**Separated/divorced**	**46.5**	**1.52**	**1.01, 2.29**	**1.23**	**0.71, 2.14**
**Widowed**	**7.1**	**2.86**	**1.56, 5.24**	**1.35**	**0.61, 2.98**
***Occupational characteristics and sexual behaviours***					
**Age at first sex (per year increase)**	**(***n* **= 1 604)**	**0.97**	**0.91, 1.03**	**-**	**-**
**Years in sex work (per year increase)**	**(***n* **= 1 592)**	**1.03**	**1.00, 1.06**	**-**	**-**
**Total monthly income (VND, using IQR categories)**	**(***n* **= 1 643)**			**-**	**-**
** < 5 million**	**-**	**1.00**	**-**	**-**	**-**
**5 to < 7 million**	**-**	**0.71**	**0.43, 1.16**	**-**	**-**
**7 to < 8.5 million**	**-**	**0.68**	**0.39, 1.17**	**-**	**-**
≥ **8.5 million**	**-**	**0.72**	**0.47, 1.10**	**-**	**-**
**Negotiate sex mainly on street or in other public areas**	**(***n* **= 1 686)** **41.1**	**1.70**	**1.14, 2.55**	**-**	**-**
**Ever sold sex in other provinces**	**(***n* **= 686)** **7.1**	**2.04**	**1.23, 3.38**	**-**	**-**
**Had over 10 sex clients in past month**	**(***n* **= 1 686)** **73.1**	**2.17**	**1.36, 3.48**	**2.16**	**1.25, 3.72**
**Consistent condom use with clients in past month**	**(***n* **= 1 686)** **71.4**	**0.99**	**0.69, 1.41**	**-**	**-**
***Alcohol and drug use behaviours***					
**Daily alcohol consumption in past month**	**(***n* **= 1 686)** **12.7**	**0.85**	**0.49, 1.48**	**-**	**-**
**Non-injection drug use (lifetime)**	**(***n* **= 1 686)** **8.9**	**3.10**	**2.01, 4.79**	**3.25**	**1.84, 5.73**
***Sex partners’ drug use behaviour***					
**Sex partners injected drugs in past month**	**(***n* **= 1 686)**	**-**	**-**	**-**	**-**
**No**	**64.9**	**1.00**	**-**	**-**	**-**
**Yes**	**11.9**	**1.51**	**0.90, 2.54**	**-**	**-**
**Unknown**	**23.2**	**1.86**	**1.30, 2.68**	**-**	**-**
***Incarceration history***					
**Ever detained in rehabilitation centre for sex workers**	**(***n* **= 1 682)** **8.02**	**2.63**	**1.64, 4.21**	**2.19**	**1.19, 4.02**
***STI***					
**Experienced genital pain or ulcers in past 12 months**	**(***n* **= 1 686)** **53.3**	**1.12**	**0.81, 1.56**	**-**	**-**
***Blood-borne infections***					
**HBV**	**(***n* **= 1 686)** **65.4**	**0.82**	**0.60, 1.11**	**-**	**-**
**HIV**	**(***n* **= 1 686)** **10.9**	**20.31**	**13.98, 29.49**	**24.16**	**14.81, 39.41**
***Province***	**(***n* **= 1 484)**	**-**	**-**	**-**	**-**
**Hanoi**	**-**	**1.32**	**0.84, 2.12**	**-**	**-**
**Hai Phong**	**-**	**1.95**	**1.34, 2.86**	**-**	**-**
**Ho Chi Minh City**	**-**	**1.00**	**-**	**-**	**-**

* *n*  **= 1828. Variables in multivariate model are adjusted for age and marital status.**

## Discussion

We found high prevalence of HCV exposure (8.8–30.4%) and active HCV infection (3.6–22.1%) among FSW across all study provinces and subpopulations, an important finding with implications for HCV prevention and treatment. The small body of studies of HCV among FSW has documented exposure but not current infection. Studies have shown lower anti-HCV prevalence ranging from 2.6% in India to 12% in China, Taiwan. (*12*, *13*) In contrast, a more recent study in Canada found higher anti-HCV seropositivity (42.5%) among FSW. (*14*)

This is the first study in Viet Nam to examine risk factors for HCV exposure among FSW. Injection drug use was the key driver of HCV infection among FSW as it is among MSM and PWID in Viet Nam. (*8*, *9*) Although only 8% of FSW reported injection drug use, almost two thirds of HCV exposures were attributable to injection drug use. Notably, HCV exposure was also high among FSW without a history of injection drug use. Non-injection drug use was a significant factor for HCV exposure among FSW. We previously found similar correlations with HIV infection among FSW, in that HIV risk increased with the use of non-injection drugs, specifically amphetamine-type stimulants, which was high in HCMC and Hanoi. (*10*) In the context of these findings, it is important that Viet Nam’s HCV interventions address FSW vulnerabilities for infection. FSW and other women who inject drugs face gender-specific health risks that lead to increased levels of injection risk behaviour, yet among PWID, women are often overlooked by harm reduction programmes. (*15*) Compulsory detention was a predictor of HCV exposure among FSW, potentially due to HCV exposure at the detention centres, or FSW engaged in higher-risk activities that exposed them to HCV and led to incarceration. Given that there is evidence of high HCV incidence in detained populations across the world, (*16*) the 2012 closure of detention centres for FSW in Viet Nam is an important policy change that can reduce the risk of HCV infection, along with HIV infection, among FSW.

The positive association of HCV exposure with having a greater number of clients indicates that FSW are vulnerable to infection through sexual risks. Although sexual intercourse has relatively low efficiency in transmitting HCV, a history of multiple sex partners may increase the probability of having sex with an infectious partner during the acute phase of infection, (*3*) and the use of stimulants potentially elevates risky sexual contact. (*17*) FSW in this study reported low levels of consistent condom use, exposing them to HIV and potentially frequent sexually transmitted infections, which increases risk for HCV infection. (*18*, *19*)

Even taking into account a history of injection drug use, HIV was independently associated with HCV, which is consistent with evidence establishing the role of viral STI in the transmission of HIV. (*20*) Since HCV and HIV share a parenteral route of transmission, the WHO-recommended approach to HIV prevention involving a comprehensive package that includes both harm reduction and condom interventions (*21*) also applies to HCV prevention programmes. Given high HCV and HIV coinfection among FSW, Viet Nam could leverage the resources and infrastructure available for HIV services to deliver HCV testing and treatment services to FSW and other key populations. HIV coinfection reduces the likelihood of spontaneous clearance of HCV infection and accelerates the progression of liver disease. (*22*)

HBV infection prevalence among FSW did not differ from that in the general population, estimated at 9.1%, (*6*) a finding that is consistent with the primarily perinatal and childhood transmission of HBV in Asia. (*23*) Prevalence of HBeAg-active HBV infections among FSW was also similar to prevalence previously detected among Vietnamese women of child-bearing age. (*24*) HBeAg positivity usually indicates viral replication and therefore high levels of infectiousness. Yet in genotypes B and C, which are most common in Viet Nam, (*25*) high viral replication can occur without the presence of HBeAg due to mutations in the precore and basal core promoter regions. The mutations suppress HBeAg synthesis while enhancing viral genome replication. (*26*, *27*) Nevertheless, as the Vietnamese Ministry of Health expands treatment, targeted provision of routine HBV testing and treatment for active HBV infection would benefit FSW and other populations where stigma and discrimination impedes access to services. For example, high antenatal screening uptake would be difficult to achieve with few sex workers presenting to health-care services. (*28*)

Increasing HCV prevalence from north to south had been expected given the older injection drug use epidemic in HCMC in the south of Viet Nam. (*29*) Instead, HCV infection was higher in the two northern provinces than in HCMC, a pattern observed also among MSM in Viet Nam. (*9*) The higher prevalence of reported injection drug use among SSW and VSW in Haiphong may explain the higher HCV infection in the north, but it contrasts with lower reported injection drug use among MSM in the north than in the south. (*9*) Further phylogenetic research may help to explain these infection patterns.

Our study has limitations, including potential misclassification of injection drug use status in that there is greater willingness to report non-injection drug use. This would lead to an overestimation of the association of HCV infection with non-injection drug use. However, our findings of additional sexual and non-injection drug use risks are consistent with studies reporting associations between HCV exposure and non-injection crack use in Canada and with increased number of sex clients and duration of sex work in China. (*14*, *30*) Another limitation was that 35 of the 271 anti-HCV-positive samples tested for HCV core antigen returned reactive results in the grey zone, which indicates possible but not confirmed active HCV infection. Almost all grey zone reactive results were from Hanoi. Inclusion of grey zone reactive results would have shown 4% and 5% higher viraemic HCV infection among Hanoi SSW and VSW, respectively. Furthermore, as a limitation of the cross-sectional survey design, the analysis could not assess temporal relationships between risk behaviour with HCV acquisition. Being able to monitor infections and risks across time would help to better target interventions, including understanding the extent to which incarceration contributed to HCV transmission.

The 2013 Vietnamese IBBS round represents the first routine integration of HCV core antigen into national viral hepatitis surveillance, of which we are aware. In contrast to most population or risk group–based estimates of HCV infection, the inclusion of HCV core antigen testing provided data on active HCV infection. HCV core antigen testing is sensitive and specific and is a reasonable alternative to HCV nucleic acid testing, providing similar accuracy without the additional logistic requirements and cost of nucleic acid testing. (*31*) Assay-based, population-based estimates of both active HCV infection and past HCV exposure are needed to forecast national prevention and diagnostic and treatment needs. In the absence of such data, estimates have been derived from external estimates through mathematic modelling of the relationship between the prevalence of active infection and exposure. As DAAs become increasingly accessible, targeting testing and treatment services to populations at higher risk of HCV infection will be the most effective way to reduce the chronic HCV morbidity and prevent further transmission.

The study showed high past and active HCV infection prevalence and HCV/HIV coinfection among Vietnamese FSW, a vulnerable population historically characterized by low HIV service uptake. Results have the potential to inform national responses designed to address dual sexual and drug-related risks for HCV infection among FSW while highlighting the need to ensure FSW access to HIV prevention and treatment services.
